# PD87 Costs of Emicizumab in Hemophilia A: Comparison Between Post-Incorporation Expenses and Government Projections in Brazil

**DOI:** 10.1017/S0266462325102146

**Published:** 2025-12-29

**Authors:** Juliana Alvares-Teodoro, Livia Nassif, Ricardo Mesquita Camelo, Augusto Guerra, Marina Silva, Luila Clicia, Barbara Santos, Francisco Acurcio

## Abstract

**Introduction:**

Hemophilia A (HA) results from mutations in the F8 gene, leading to a deficiency in factor VIII (FVIII) and consequent spontaneous bleeds. Treatment options include bleeding prophylaxis with FVIII and, more recently, emicizumab (EMI), a FVIII-mimetic bispecific antibody. EMI was introduced into the Brazilian Unified Health System in 2019 for people with HA and inhibitors (PwHAi). This study compared EMI costs with prior prophylactic methods and evaluated its budgetary impact against government projections.

**Methods:**

The National Registry of People with HA Using Emicizumab in Brazil (EMCase) is a prospective observational study initiated in 2020, conducted in 16 Brazilian hemophilia treatment centers. The analysis included consumption of FVIII, bypassing agents, and EMI. Treatment costs for the pre-EMI period and the first year of EMI use were calculated using prices paid by the Ministry of Health, as reported in the Brazilian Health Price Database. Yearly average treatment costs per kilogram of body weight were compared with government budget projections.

**Results:**

A total of 56 moderate to severe PwHAi were included. Before EMI treatment, the actual treatment costs were BRL31,145.19 (USD5,190.87) per kg per year. During the first year of EMI prophylaxis, the actual treatment costs were BRL22,583.79 (USD3,763.96) per kg per year, representing approximately 27.5 percent reduction in costs, compared with the previous period. In addition, the government projected cost for 2019, adjusted for inflation, was estimated at BRL19,736.84 (USD3,289.47) per kg per year for the first year of EMI use, resulting in a 14.4 percent increase in the projected budget impact.

**Conclusions:**

Although the actual cost during the first year of EMI prophylaxis was higher than the government project cost, a cost reduction was observed in comparison with the pre-EMI period cost. The study highlighted the importance of real-world data-based economic analyses in the evaluation of health technologies, complementing government projections to inform decision-making.

